# *Coral dealbatus* Crude Polysaccharide Attenuates Fat Accumulation and Intestinal Flora Disorders in Mice Fed with a High-Fat Diet

**DOI:** 10.3390/foods14213734

**Published:** 2025-10-30

**Authors:** Yan Shen, Jianyang Fu, Jinya Dong, Zezhu Du, Jun He, Yuanfeng Chen, Siyu Zhou, Huiqing Luo, Shengjie Duan, Linxian Shan, Jingchuan Zheng, Xiaocui Du, Yunfei Ge, Chongye Fang, Ruijuan Yang

**Affiliations:** 1 College of Food Science and Technology, Yunnan Agricultural University, Kunming 650201, China; 18184494648@163.com (Y.S.); 15068157092@163.com (J.F.); dongjinya@163.com (J.D.); pj18213743420@163.com (Z.D.); hjun202203@163.com (J.H.); 19184056918@163.com (Y.C.); zzz121300y@163.com (S.Z.); 18788107018@163.com (H.L.); w2414510451@163.com (S.D.); slx90895@163.com (L.S.); 18788190140@163.com (J.Z.); 18345965861@163.com (Y.G.); 2 College of Agronomy and Biotechnology, Yunnan Agricultural University, Kunming 650201, China; 2010017@ynau.edu.cn

**Keywords:** *Coral dealbatus*, crude polysaccharide, fat accumulation

## Abstract

Obesity, a major risk factor for cardiometabolic diseases, arises from chronic energy imbalance and ectopic lipid deposition. This study investigated the anti-obesity potential of *Coral dealbatus* crude polysaccharides (CDP), a previously uncharacterized bioactive fraction from a hybrid vegetable cultivar developed by the Chinese Academy of Agricultural Sciences. CDP, obtained via hydroalcoholic extraction, was structurally characterized as amorphous with heterogeneous molecular weights (87,813 Da, 4158 Da, and 728 Da) and glucose-dominant monosaccharide composition (FT-IR, XRD, and HPLC). In a high-fat diet (HFD)-induced murine obesity model, oral CDP administration significantly attenuated body weight gain (*p* < 0.05) and reduced ectopic lipid deposition. Histopathological analysis confirmed CDP’s efficacy in ameliorating hepatic steatosis, evidenced by diminished lipid droplet accumulation. Furthermore, CDP reversed HFD-induced gut microbiota dysbiosis, modulating beneficial bacterial taxa. These findings demonstrate CDP’s therapeutic potential against diet-induced metabolic disorders, likely mediated through lipid metabolism regulation and intestinal microbiota modulation, supporting its development as a novel functional food ingredient for dietary intervention.

## 1. Introduction

Obesity, a metabolic disorder of increasing prevalence, has become a major global health challenge. It is characterized by pathological adipose accumulation and systemic metabolic dysregulation and is associated with increased risk of multiple comorbidities. Recent epidemiological data indicate substantial increases in obesity-associated conditions, including type 2 diabetes mellitus, cardiovascular disease, hypertension, and obstructive sleep apnea [1,2]. The primary driver of obesity is chronic energy imbalance, in which energy intake persistently exceeds expenditure, leading to ectopic lipid deposition across multiple tissues [3]. Although genetic background and endocrine disorders confer susceptibility, the principal modifiable determinants are sustained consumption of energy-dense diets and physical inactivity/sedentary behavior.

Individuals with obesity commonly exhibit reduced intestinal microbial diversity, characterized by lower abundances of putatively beneficial commensals, increased pathobiont taxa, and altered microbial metabolites. Such dysbiosis may further exacerbate host metabolic dysregulation, creating a self-reinforcing cycle. Accordingly, modulation of gut microbiota structure and function is increasingly recognized as a therapeutic strategy for obesity and related metabolic complications. Consistent with this concept, numerous studies have shown that bioactive natural extracts ameliorate high-fat diet (HFD)-induced obesity in murine models, at least in part by modulating the gut microbiota. Reported examples include *Ganoderma amboinense* polysaccharide [4], Caffeic acid [5], and Allicin [6].

*Coral dealbatus* is a functional vegetable cultivar developed by the Chinese Academy of Agricultural Sciences via interspecific hybridization between the rare wild Feicai (*Sedum aizoon* L.) and Jingtian Sanqi (*Gynura japonica*). This hybrid is primarily adapted to, and cultivated in, northern, central, and eastern China. Despite its agronomic potential, commercial cultivation remains limited, and existing research focuses mainly on agronomic practices and postharvest processing, with relatively few studies addressing its pharmacological properties [7]. Polysaccharides are natural biomacromolecules extractable from diverse sources, including plants [8], and they exhibit a broad spectrum of biological activities [9]. For example, polysaccharides from *Acanthopanax* ameliorate alcoholic fatty liver by regulating the gut–liver axis [10]; pumpkin polysaccharides display antidiabetic effects in type 2 diabetic rats [11]; *Auricularia auricula* polysaccharides alleviate non-alcoholic fatty liver [12], *Poria cocos* polysaccharides exert immunomodulatory effects; and ginger polysaccharides show anti-fatigue activity [13]. *Coral dealbatus* crude polysaccharides (CDP) refer to the crude polysaccharide fraction extracted from *Coral dealbatus*. However, it remains unclear whether CDP can attenuate fat accumulation in mice fed a high-fat diet (HFD).

*Coral dealbatus* is a plant with recognized medicinal and nutritional value and substantial potential for research and development. Reported bioactive constituents include marigold glucosides, oleanolic acid, and flavonoids. In traditional medicine, the plant is attributed with functions such as “nourishing the heart and liver,” “promoting blood circulation,” and “clearing heat and toxins”; contemporary studies further suggest potential benefits in the prevention and management of cardiovascular and cerebrovascular conditions. As a nutritious vegetable, *Coral dealbatus* can be processed into a variety of products with considerable market potential. From an economic perspective, large-scale cultivation may increase farmers’ income. The species also exhibits broad environmental adaptability and has been considered for agricultural production and ecological restoration. Reflecting these considerations, *Coral dealbatus* has been cultivated on a large scale in Nujiang Prefecture, Yunnan Province (China), as part of poverty alleviation programs, where it contributes to local household income. Nevertheless, peer-reviewed studies on *Coral dealbatus* remain limited and have focused primarily on cultivation and post-harvest practices, leaving its biological activities and functional properties under-investigated. Building on preliminary work, we extracted crude polysaccharides from *Coral dealbatus* (CDP) and evaluated their effects on obesity in high-fat diet (HFD) fed mice and on the gut microbiota, with the aim of addressing this knowledge gap and informing value-added utilization relevant to regional development.

In this study, crude polysaccharides from *Coral dealbatus* (CDP) were obtained by hydroalcoholic extraction followed by ethanol precipitation. We established a high-fat diet (HFD)-induced murine model of obesity to evaluate the effects of CDP on metabolic and histological endpoints relevant to diet-induced obesity. This work is intended to provide a preclinical basis to inform the development of CDP-based functional ingredients and related natural products.

## 2. Materials and Methods

### 2.1. Materials and Reagents

Dried *Coral dealbatus* material was purchased from Lushui Jianglin Poverty Alleviation Professional Cooperative (Lushui, Yunnan, China). Assay kits for total cholesterol (TC), triglycerides (TG), high-density lipoprotein cholesterol (HDL-C), low-density lipoprotein cholesterol (LDL-C), alanine aminotransferase (ALT), aspartate aminotransferase (AST), and bicinchoninic acid (BCA) protein quantification were purchased from Nanjing Jiancheng Bioengineering Institute (Nanjing, Jiangsu, China).

### 2.2. Preparation of CDP

CDP was prepared according to a published method with minor modifications [14]. Briefly, dried *Coral dealbatus* material was milled to a fine powder and extracted with deionized water at a material-to-liquid ratio of 1:10 (*w*/*v*) in three successive batches (30 min each), and the three filtrates were combined and concentrated. Absolute ethanol was then added to a final concentration of 70% (*v*/*v*). The mixture was kept at 4 °C overnight (~12 h) to precipitate polysaccharides, and the resulting precipitate was collected. The precipitate was redissolved in deionized water, deproteinized by the Sevag method (chloroform/n-butanol = 4:1, *v*/*v*), and lyophilized to obtain CDP.

### 2.3. Determination of Physicochemical Indexes of CDP

The total carbohydrate content of CDP was determined by the phenol–sulfuric acid method [15], protein content was measured using the bicinchoninic acid (BCA) assay; total flavonoid content was quantified according to SN/T 4592-2016 [16]; and total phenolic content was determined according to T/NAIA 097-2021 [17].

### 2.4. Preliminary Characterization of CDP

#### 2.4.1. Electron Microscope Scanning

Samples were mounted on metal stubs, sputter-coated with a thin gold film, and imaged using a scanning electron microscope (GeminiSEM 360, Zeiss, Obercohen, Germany).

#### 2.4.2. Infrared Spectral Analysis and X-Ray Polycrystalline Diffraction Analysis

CDP powder was intimately mixed with spectroscopic-grade potassium bromide (KBr), finely ground, and pressed into pellets. Spectra were analyzed on a Fourier transform infrared (FT-IR) spectrometer (IRTracer-100, Shimadzu, Kyoto, Japan) over 4000–400 cm^−1^.

Powder crystallinity was examined by X-ray diffraction (XRD). Measurements were performed on a powder X-ray diffractometer (D8 Advance, Bruker, Karlsruhe, Germany) operated at 40 kV and 30 mA. Data were collected over 2θ = 10–80° at a scan rate of 5°·min^−1^ to obtain the crystallographic profile of CDP.

#### 2.4.3. Analyzing the Molecular Weight and Monosaccharide Composition

The molecular weight and distribution of CDP were analyzed using an Agilent 1260 Infinity II liquid chromatography system (Santa Clara, CA, USA) equipped with a refractive index detector (RID). Column: PL aquage 1-OH MIXED-H 8 μm 300 × 7.5 mm; detector: RID; temperature: 40 °C; mobile phase: 0.1 mol/L NaNO_3_; injection volume: 20 μL; flow rate: 1 mL/min. The system was calibrated using a series of dextran standards with known molecular weights to plot a standard curve of retention time versus the logarithm of molecular weight (logMW). The molecular weight distribution of the sample was automatically calculated using the workstation software based on this calibration curve.

The monosaccharide composition was detected using Shimadzu LC-20AD (Japan). Column: Xtimate C18 4.6 × 200 mm 5 um; mobile phase: 0.05 M potassium dihydrogen phosphate solution; injection volume: 20 μL; column temperature: 30 °C; flow rate: 1.0 mL/min.

### 2.5. Animal Experiment

**Figure 3:**
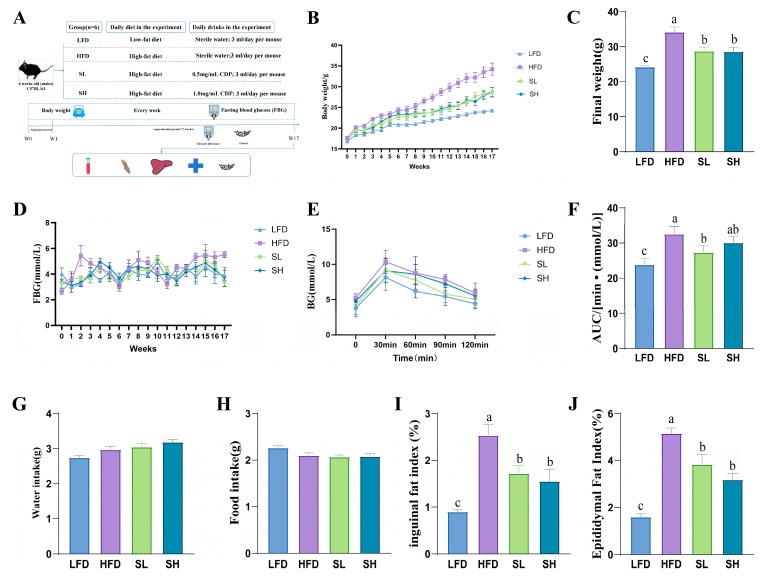
The effect of CDP on growth indices in mice. (**A**) Animal experimental design; (**B**) body weight curves; (**C**) final body weight; (**D**) weekly fasting blood glucose curves; (**E**,**F**) glucose tolerance curves and area under the curve; (**G**) average daily water intake; (**H**) average daily food intake; (**I**) inguinal adiposity coefficients; and (**J**) epididymal adiposity coefficients. Data are expressed as Mean ± SEM. Different letters (a, b, and c) indicate significant differences between different experimental groups (*p* < 0.05).

Specific Pathogen-Free (SPF) male C57BL/6J mice (6-week-old) were obtained from Henan Skibbes Biotechnology Co., Ltd. (Anyang, Henan, China). The mice were housed in the animal facility of Yunnan Agricultural University under controlled conditions (12 h light/12 h dark cycle; 22–24 °C) in individually ventilated cages (IVC). All animal procedures complied with the Guide for the Care and Use of Laboratory Animals and were approved by the Life Science Ethics Committee of Yunnan Agricultural University (Approval No. 202502002). After a 1-week acclimation, the mice were randomly assigned to four groups (n = 6/group): (i) low-fat diet control (LFD), fed standard chow and given sterile water; (ii) high-fat diet model (HFD), fed an HFD and given sterile water; (iii) low-dose CDP (SL), HFD-fed and given Coral dealbatus crude polysaccharide (CDP) at 0.5 mg/mL in drinking water; and (iv) high-dose CDP (SH), HFD-fed and given CDP at 1.0 mg/mL in drinking water. CDP or sterile water was provided ad libitum via the drinking water system. Body weight and fasting blood glucose (FBG) were measured weekly. An oral glucose tolerance test (OGTT) was performed at week 15. At week 16, fecal samples were collected and stored at −80 °C for subsequent analyses. At the end of week 17, mice were euthanized. Blood and key organs (e.g., liver, adipose tissue) were collected for biochemical assays and histopathological examination. The experimental timeline is summarized in Figure 3A.

### 2.6. Human-Equivalent Dose (HED) Calculation (Methods)

To facilitate translation, body surface area scaling was used (Km: mouse = 3, human = 37) [18]. C57BL/6J mice were assumed to weigh 30 g with a daily water intake of 3 g (water density ≈ 1 g/mL). The mouse dose was calculated as follows:

$$D_{\mathrm{mouse}}(\mathrm{mg}\backslash {\mathrm{kg}}^{-1}\;\backslash \;{\mathrm{day}}^{-1})=\frac{C\times V}{\mathrm{BW}}$$

where *C* is CDP concentration in drinking water (mg·mL^−1^), *V* = 3 mL·day^−1^, and *BW* = 0.03 kg. Thus, 0.5 mg·mL^−1^ → 50 mg·kg^−1^·day^−1^; 1.0 mg·mL^−1^ → 100 mg·kg^−1^·day^−1^

The human-equivalent dose (HED) was as follows:

$$\mathrm{HED}(\mathrm{mg}\backslash {\mathrm{kg}}^{-1}\;\backslash \;{\mathrm{day}}^{-1})=D_{\mathrm{mouse}}\times 373$$

yielding 4.05 and 8.11 mg·kg^−1^·day^−1^. Total human doses were 243–284 mg·day^−1^ and 486–568 mg·day^−1^ for 60–70 kg adults. These computations provide translational context and were not used for hypothesis testing.

### 2.7. Serum Biochemical Analysis

After collection, whole blood was allowed to stand at 4 °C for 1 h and was then centrifuged at 10,000 rpm for 10 min in a refrigerated centrifuge. The serum was collected and stored at −80 °C until analysis. Serum TC, TG, HDL-C, LDL-C, ALT, and AST were measured using commercial assay kits according to the manufacturer’s instructions.

### 2.8. Liver Biochemical Indicators

Liver tissue (~50 mg) was accurately weighed, and anhydrous ethanol was added at a tissue-to-solvent ratio of 1:9 (*w*/*v*; e.g., 50 mg in 0.45 mL). Samples were mechanically homogenized in an ice–water bath and centrifuged at 2500 rpm for 10 min at 4 °C. The supernatant was collected for analysis. Total cholesterol (TC) and triglycerides (TG) in the supernatant were measured immediately using commercial assay kits (Nanjing Jiancheng Bioengineering Institute, China) according to the manufacturer’s instructions.

### 2.9. Histopathological Analysis

Epididymal white adipose tissue (eWAT) and liver were collected from mice, fixed in 4% paraformaldehyde for at least 24 h, processed, and embedded in paraffin. Paraffin sections (4 μm) were stained with hematoxylin and eosin (H&E). For neutral lipid visualization, fresh-frozen liver cryosections (8–10 μm) were stained with Oil Red O and counterstained with hematoxylin.

### 2.10. Analysis of Intestinal Flora

#### 2.10.1. Sample Collection

Mouse fecal samples stored at −80 °C were removed (n = 3).

#### 2.10.2. DNA Extraction

Microbial genomic DNA was extracted from mouse intestinal samples using the E.Z.N.A.^®^ Mag-Bind Soil DNA Kit (Omega Bio-tek, Norcross, GA, USA; catalog No. M5635-02) in accordance with the manufacturer’s instructions. DNA concentration was quantified with a Qubit 4 Fluorometer (Thermo Fisher Scientific, Waltham, MA, USA), and samples with concentrations ≥10 ng/μL were retained for downstream analyses. DNA purity was assessed by UV–Vis spectrophotometry, and only samples with an A260/A280 ratio of 1.8–2.0 were accepted.

#### 2.10.3. PCR Amplification of 16S rRNA Gene

The hypervariable V3–V4 region of the bacterial 16S rRNA gene was amplified using 2 × Hieff^®^ Robust PCR Master Mix (Yeasen, Shanghai, China; cat. 10105ES03). PAGE-purified universal primers 341F (5′-CCTACGGGGNGGCWGCAG-3′) and 806R (5′-GACTACHVGGGGTATCTAATCC-3′) were used. PCRs were set up in 30 μL containing 15 μL 2 × Master Mix, 2 μL template DNA (10 ng/μL), 1 μL each of 10 μM forward and reverse primers, and nuclease-free water to volume. Cycling conditions: initial denaturation at 95 °C for 3 min; 5 touchdown cycles of 95 °C for 30 s, 45 °C for 30 s, and 72 °C for 30 s; followed by 20 cycles of 95 °C for 30 s, 55 °C for 30 s, and 72 °C for 30 s; and a final extension at 72 °C for 5 min. Amplicons were verified on 2% agarose gels (TBE) stained with ethidium bromide and purified using Hieff NGS™ DNA Selection Beads (Yeasen, China) according to the manufacturer’s protocol.

#### 2.10.4. 16S Gene Library Construction, Quantification, and Sequencing

Library construction was performed by Bioengineering (Shanghai, China) Co., Ltd. using Illumina-compatible universal adapters and index sets. Library quality was assessed using a Qubit^®^ 4 Fluorometer with the dsDNA HS assay (Thermo Fisher Scientific, USA) and an Agilent 2100 Bioanalyzer (Agilent, Santa Clara, CA, USA). Equimolar-pooled libraries were sequenced on an Illumina MiSeq platform (Illumina, San Diego, CA, USA) using a 2 × 300 bp paired-end strategy.

#### 2.10.5. Sequence Processing, OTU Clustering, Representative Tag Matching, and Biological Classification

Paired-end reads were merged with PEAR (v0.9.8) and clustered into operational taxonomic units (OTUs) at 97% sequence similarity using USEARCH (v11.0.667). Bacterial and fungal taxonomic assignments were performed against the RDP database (release 11.5) and the UNITE ITS database (v8.2), respectively; chimeric sequences and singleton OTUs were identified and removed.

#### 2.10.6. Statistical Analyses

Alpha-diversity (Shannon and Simpson indices) was computed in mothur (v3.8.31). Rarefaction and rank-abundance curves were generated with the vegan package (v2.5-6) in R (v3.6.0). β-Diversity was assessed by principal coordinates analysis (PCoA) and non-metric multidimensional scaling (NMDS); between-group differences were evaluated by PERMANOVA. Differential features were identified using LEfSe (linear discriminant analysis effect size) with α = 0.05 and an LDA score threshold, and, where appropriate, group comparisons were performed in STAMP (v2.1.3).

#### 2.10.7. Functional Prediction

For functional prediction, metabolic pathway prediction was performed based on 16S rRNA gene data using PICRUSt (v1.1.4) compared to the KEGG database (Release 94.0). All statistical analyses were implemented using SPSS 22.0, and the error lines of the visualization charts indicate 95% confidence intervals.

### 2.11. Correlation Analysis

In this study, multivariate correlation analysis was used to systematically examine associations between the gut microbiota OTU abundance matrix and host physiological indices across experimental and control groups. Physiological indices included relative organ weights (liver, inguinal fat, and epididymal fat; organ weight/final body weight), final body weight, and four blood lipid measures—total cholesterol (TC), triglycerides (TG), high-density lipoprotein cholesterol (HDL-C), and low-density lipoprotein cholesterol (LDL-C). During preprocessing, organ indices were standardized as organ wet weight (g)/final body weight (g), and lipid concentrations were harmonized to mmol/L to ensure comparability. Using Bray–Curtis dissimilarities for microbiota profiles and Euclidean distances for physiological indices, we first applied the Mantel test to assess global associations between community structure and host physiology. When significant global associations were detected, we further evaluated concordance between microbial community structure and host phenotypes using Procrustes analysis, thereby assessing the spatial alignment of the two ordinations.

### 2.12. Statistical Analysis

All experimental data were at least three replicated samples, and the results were expressed as mean ± standard error (SEM). One-way ANOVA followed by post hoc Duncan’s test was performed using SPSS 22.0 software to compare the mean differences between the experimental groups, and *p* < 0.05 was considered statistically significant. Graphs were generated using GraphPad Prism 7.0 software and Origin 2024 software.

## 3. Results

### 3.1. Physicochemical Properties of CDP

The compositional analysis of the crude polysaccharide fraction (CDP) revealed the following percentages by dry weight (mean ± SEM): total carbohydrates at 42.01 ± 0.45%, proteins at 6.89 ± 0.82%, total flavonoids at 0.36 ± 0.03%, and total phenolics at 8.85 ± 0.07%. The extraction yield of CDP from dried *Coral dealbatus* material was 5.20 ± 0.17% (*w*/*w*, dry weight).

### 3.2. Preliminary Characterization of CDP

#### 3.2.1. Electron Microscope Scanning

**Figure 1:**
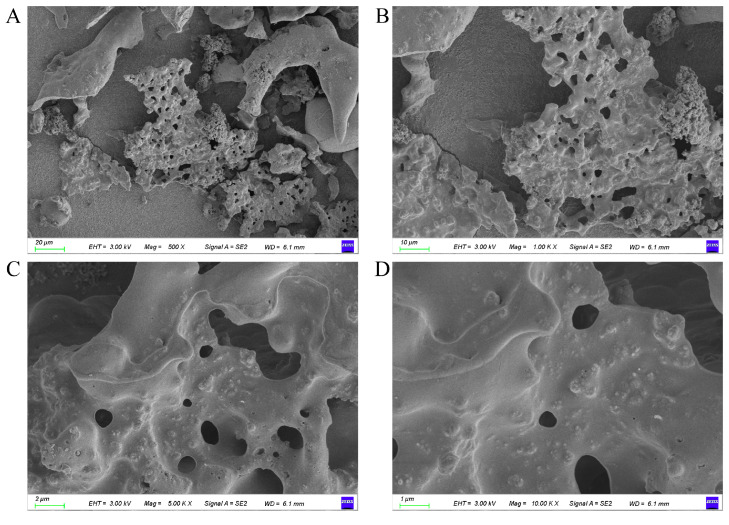
SEM analysis of CDP. (**A**) 500×; (**B**) 1.00K×; (**C**) 5.00K×; (**D**) 10.00K×.

As shown in Figure 1, low-magnification SEM micrographs of CDP (Figure 1A,B) revealed polygonal particles with numerous, loosely distributed surface voids. At higher magnification (Figure 1C,D), the particles displayed an irregular lamellar morphology with surface protrusions composed of fine particulates and occasional elliptical pores.

#### 3.2.2. Infrared Spectral Analysis and X-Ray Polycrystalline Diffraction Analysis

**Figure 2:**
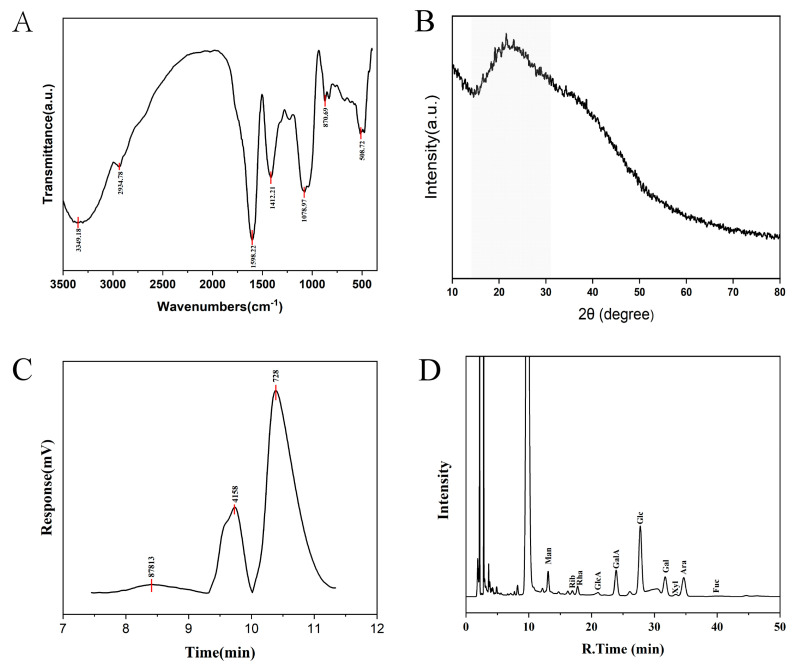
Analysis and identification of CDP. (**A**) FT-IR analysis of CDP; (**B**) XRD analysis of CDP; (**C**) GPC chromatogram for CDP; (**D**) monosaccharide composition analysis of CDP.

Functional groups in CDP were identified by FT-IR spectroscopy (Figure 2A). The broad band at 3349 cm^−1^ corresponds to O–H stretching, indicating abundant hydroxyl groups, and the band at 2935 cm^−1^ is attributed to aliphatic C–H stretching (–CH_2_/–CH_3_). The band at 1598 cm^−1^ likely arises from the antisymmetric stretching of carboxylate (COO^−^) groups—consistent with uronic acid residues (e.g., galacturonic or mannuronic acid)—whereas the band at 1412 cm^−1^ corresponds to symmetric COO^−^ stretching and/or C–H bending. Additionally, a strong band near 1079 cm^−1^ is characteristic of C–O–C and C–O stretching vibrations associated with glycosidic linkages in the polysaccharide backbone.

As shown in Figure 2B, the CDP consists of two different peaks appearing at 2θ values of 14.876° and 21.536°, but the overall peaks are not obvious, which may be produced by amorphous substances, and combined with the SEM analysis, it can be seen that there is no obvious crystal structure of the CDP, which indicates that the CDP belongs to the amorphous property.

#### 3.2.3. Analysis of Molecular Weight and Monosaccharide Composition

In order to study the biological activity of CDP, the molecular weight and monosaccharide composition of CDP were analyzed, and the molecular weight analysis showed that the main molecular weights of CDP were 87,813 Da, 4158 Da, and 728 Da, indicating that CDP is a polysaccharide that exists in a mixture of high molecular weights and low molecular weights (Figure 2C).

CDP contains a variety of monosaccharides, including mannose, ribose, rhamnose, glucuronic acid, galacturonic acid, glucose, galactose, xylose, arabinose, and fucose. The most abundant were glucose (45.197 μg/mL) and galacturonic acid (17.011 μg/mL), as shown in Table 1.

### 3.3. Effect of CDP on Body Weight and Organ Index of Mice

The change curve of mouse body weight is shown in Figure 3B, and it can be seen that the trend of body weight growth of mice in the HFD group was significantly higher than that of other experimental groups. The final body weight of the LFD group was extremely significantly lower than that of the HFD group (*p* < 0.01), which proved that the establishment of high-fat model obese mice was successful, and the body weights of mice in the SL and SH groups were significantly lower than that of the HFD group (*p* < 0.05), which proved that CDP had a certain mitigating effect on body weight growth induced by a high-fat diet in the mice (Figure 3C). CDP did not affect the amount of water intake (Figure 3G) and the amount of food intake (Figure 3H) in the mice (*p* > 0.05). The area under the glucose tolerance curve in the SL group was significantly lower than that in the HFD group (*p* < 0.05), demonstrating that low-dose CDP alleviated glucose intolerance induced by the high-fat diet in mice (Figure 3E,F). CDP had no effect on fasting glucose in mice (Figure 3D) (*p* > 0.05). CDP significantly reduced the inguinal adiposity coefficients of the mice fed a high-fat diet (Figure 3I) and epididymal fat coefficient (Figure 3J) (*p* < 0.05).

### 3.4. Effects of CDP on Serum Biochemical Indexes in Mice

**Figure 4:**
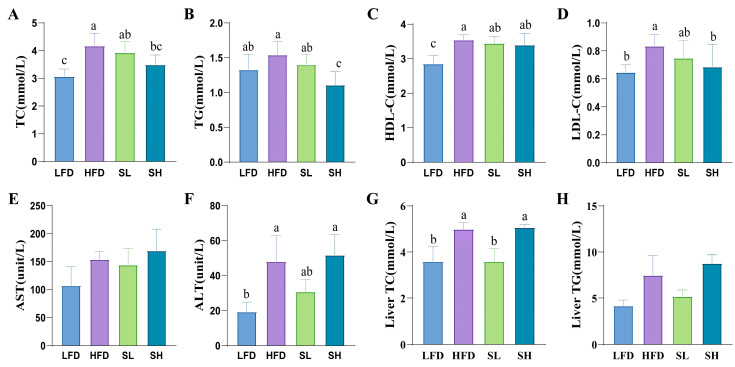
The effects of CDP on biochemical indices in mice. (**A**) serum TC level; (**B**) serum TG level; (**C**) serum HDL-C level; (**D**) serum LDL-C level; (**E**) serum AST level; (**F**) serum ALT level; (**G**) the effect of CDP on hepatic TC level; (**H**) the effect of CDP on hepatic TG level. Data are expressed as Mean ± SEM. Different letters (a, b, and c) indicate significant differences between different experimental groups (*p* < 0.05).

The high dose of CDP effectively reduced the serum levels of TC (Figure 4A), TG (Figure 4B), and LDL-C (Figure 4D) in mice on a high-fat diet (*p* < 0.05). In HDL-C (Figure 4C), AST (Figure 4E), and ALT (Figure 4F) levels, CDP did not show a better effect (*p* > 0.05).

### 3.5. Effects of CDP on Liver Biochemical Indexes in Mice

As shown in Figure 4G,H, *Coral dealbatus* crude polysaccharides (CDP) modulated hepatic total cholesterol (TC) but not triglyceride (TG) levels in mice. Compared to the low-fat diet control (LFD) group, the high-fat diet (HFD) group exhibited significantly elevated hepatic TC levels (*p* < 0.05). Notably, supplementation with the low dose of CDP (SL group) significantly reduced hepatic TC levels in HFD-fed mice (Figure 4G). In contrast, CDP administration did not significantly affect hepatic TG levels in either dose group (Figure 4H).

### 3.6. Effect of CDP on Histopathology of Adipose and Liver in Mice

**Figure 5:**
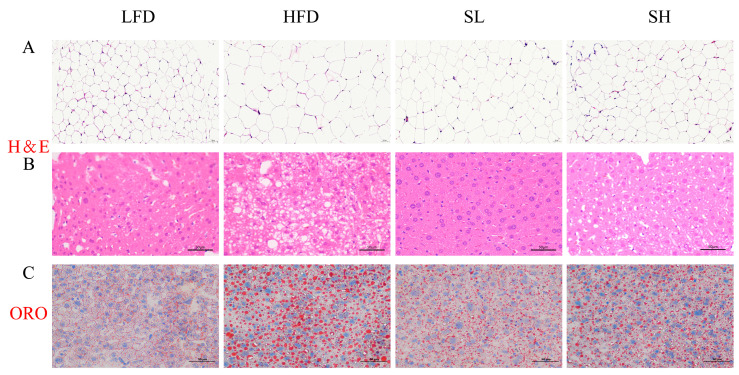
The effect of CDP on histopathology in mice. (**A**) H&E staining of epididymal fat; (**B**) H&E staining of liver; (**C**) Oil red O staining of liver. LFD: low-fat diet group; HFD: high-fat diet group; SL: CDP low-dose group; SH: CDP high-dose group.

Histopathological analyses indicated that CDP supplementation ameliorated high-fat diet (HFD)–induced alterations in epididymal adipose tissue and liver. In epididymal adipose tissue, hematoxylin–eosin (H&E) staining showed uniform adipocyte morphology and normal cell size in the low-fat diet (LFD) group, whereas the HFD group exhibited marked adipocyte hypertrophy. Both low-dose (SL) and high-dose (SH) CDP partially attenuated HFD-induced adipocyte enlargement (Figure 5A). Analysis of liver sections further corroborated these observations. H&E staining revealed preserved hepatic architecture with neatly arranged hepatocytes in the LFD and SL groups, whereas the HFD group displayed extensive macrovesicular steatosis—evidenced by large cytoplasmic lipid vacuoles—and disrupted cellular organization (Figure 5B). Oil Red O staining of neutral lipids confirmed substantial lipid droplet accumulation within hepatocytes in the HFD group, in contrast to the minimal deposition observed in the LFD, SL, and SH groups (Figure 5C). Collectively, these findings indicate that CDP mitigates HFD-induced ectopic lipid accumulation in adipose tissue and liver.

### 3.7. Effect of CDP on Intestinal Microorganisms in Mice

**Figure 6:**
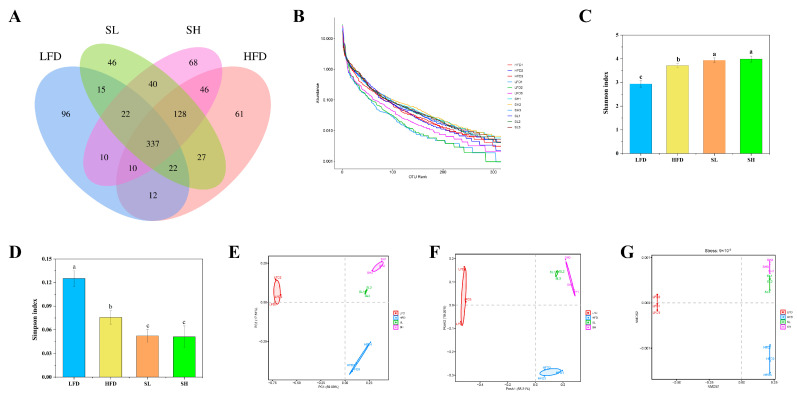
The effect of CDP on gut microbial richness in mice. (**A**) OTU_Venn; (**B**) rank_abundance; (**C**) Shannon index; (**D**) Simpson index; (**E**) PCA score plot; (**F**) PCoA score plot; (**G**) NMDS map. Data are expressed as Mean ± SEM. Different letters (a, b, and c) indicate significant differences between different experimental groups (*p* < 0.05).

Analysis of gut microbiota composition revealed significant differences among experimental groups (Figure 6). The number of unique microbial species was highest in the LFD group (96 species), followed by the HFD (61 species), SH (68 species), and SL (46 species) groups (Figure 6A). Rank abundance curves further illustrated community structures (Figure 6B). Alpha-diversity analysis demonstrated that the HFD group exhibited a significantly lower Shannon index compared to both the LFD group (*p* < 0.05) and the CDP-treated groups (SL and SH, *p* < 0.05) (Figure 6C). Conversely, the Simpson index was significantly higher in the LFD group than in all other groups (HFD, SL, SH; *p* < 0.05). Beta-diversity assessment using Principal Component Analysis (PCA, Figure 6E) indicated distinct clustering of the LFD group samples, while samples from HFD, SL, and SH groups showed greater similarity to each other. Principal Coordinates Analysis (PCoA, Figure 6F) confirmed significant overall differences in community composition between groups, with the exception of minimal separation between SL and SH groups. Non-metric Multidimensional Scaling (NMDS, Figure 6G) analysis further highlighted substantial dissimilarity between the HFD and SH groups.

**Figure 7:**
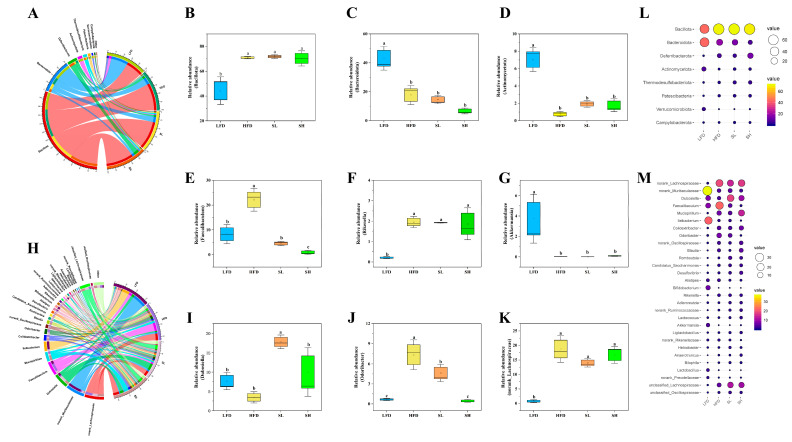
The effect of CDP on mouse intestinal flora at the phylum and genus levels. (**A**) Phylum-level chord diagram; (**B**) relative abundance of Bacillota; (**C**) relative abundance of Bacteroidota; (**D**) relative abundance of Actinomycetota; (**E**) relative abundance of *Faecalibaculum*; (**F**) relative abundance of *Rikenella*; (**G**) relative abundance of *Akkermansia*; (**H**) genus-level chord diagram; (**I**) relative abundance of *Dubosiella*; (**J**) relative abundance of *Odoribacter*; (**K**) relative abundance of norank_Lachnospiraceae; (**L**) phylum-level bubble plot of taxon distribution; (**M**) genus-level bubble plot of taxon distribution. Data are expressed as Mean ± SEM. Different letters (a, b, and c) indicate significant differences between different experimental groups (*p* < 0.05).

Taxonomic profiling revealed that dietary intervention induced significant restructuring of the gut microbiota at both the phylum and genus levels (Figure 7). At the phylum scale (Figure 7A–D), the LFD group exhibited a marked reduction in Bacillota (*p* < 0.05) and a concomitant expansion of Bacteroidota and Actinomycetota (*p* < 0.05) relative to the HFD, SL, and SH groups. Genus-level analysis (Figure 7H–K) further delineated these shifts: *Faecalibaculum* was significantly depleted in the LFD and SL groups (*p* < 0.05) and further diminished in the SH group (*p* < 0.01), whereas *Dubosiella* was selectively enriched in the SL group (*p* < 0.05). *Odoribacter* displayed a progressive decline, being significantly reduced in the SL group (*p* < 0.05) and highly significantly reduced in the SH group (*p* < 0.01). Although *Rikenella*, *Akkermansia*, and an unclassified member of the Lachnospiraceae (Figure 7F,G,K) showed no statistically significant inter-group differences under the present criteria, the overall genus-level patterns were consistent with the species distribution bubble plots (Figure 7L–M).

**Figure 8:**
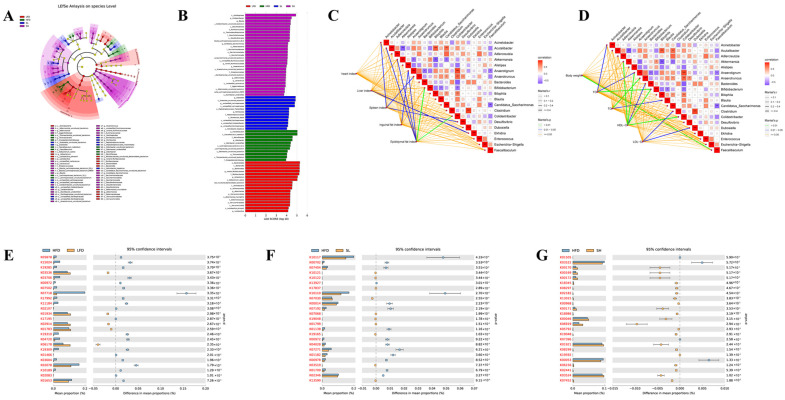
Mouse intestinal flora and correlation analysis. (**A**) Circular dendrogram of LEFSe analysis at species level; (**B**) histogram of LEFSe analysis at species level; (**C**) heat map of the network of correlation between organ index and intestinal flora; (**D**) heat map of the network of correlation between the four items of lipids and intestinal flora; and (**E**–**G**) plots of the results of the analysis of the KEGG difference.

Microbial profiling identified six orders and 10 families across all samples, and LEfSe (LDA score > 4) detected 20 discriminative genera (Figure 8A). Taxon-specific LEfSe results showed group-dependent enrichment: Bacteroidota and Akkermansiaceae were enriched in the LFD group; Faecalibaculum and Odoribacter in the HFD group; Dubosiella and Romboutsia in the SL group; and Anaerotribacter, Colidextribacter, and Blautia in the SH group (LDA > 4, Figure 8B). Host–microbiota correlations were evident: the epididymal fat index correlated with Colidextribacter and Bacteroides abundance (Figure 8C); final body weight correlated with Faecalibaculum; serum total cholesterol (TC) with Bifidobacterium; triglycerides (TG) with *Anaerotruncus*; and HDL-C with *Akkermansia*, *Anaerotignum*, *Bifidobacterium*, and *Colidextribacter* (Figure 8D). Predicted functional profiles (Table 2) differed among the groups. Relative to HFD, the LFD group showed higher predicted abundances of the ribonuclease *p* protein component and 5-formyltetrahydrofolate cyclo-ligase, and lower abundance of the two-component sensor histidine kinase YesM (Figure 8E); The SL group exhibited decreased predicted abundances of the raffinose/stachyose/melibiose transport-system substrate-binding and permease proteins (Figure 8F); whereas the SH group showed reduced representation of the electron transfer flavoprotein alpha subunit and ketol-acid reductoisomerase (Figure 8G).

## 4. Discussion

Obesity is a chronic condition characterized by excessive adipose accumulation that leads to body weight or adiposity exceeding healthful ranges, thereby increasing health risks [19]. Natural extracts are valued for bioactivity, natural origin, and functional diversity and are often regarded as having favorable safety and environmental profiles compared with some synthetic compounds, although outcomes are context-dependent. Bioactive extracts have shown promise in geroprotection, glycemic control [20], cardiovascular health [21], and obesity management [22]. In this study, crude polysaccharides from *Coral dealbatus* (CDP) were characterized by compositional assays, scanning electron microscopy (SEM), FT-IR, XRD, and monosaccharide profiling. Within CDP, total carbohydrate and protein contents were 42.01 ± 0.45% and 6.89 ± 0.82% (*w*/*w*, dry weight; mean ± SEM), respectively. At the preliminary screening stage, focusing on crude extracts is cost-effective; however, elucidating the structures of active constituents is essential for mechanistic insight and translational development. Accordingly, based on the activity observed here, we plan bioactivity-guided fractionation—graded precipitation, cellulose-based column chromatography, and gel filtration—to isolate highly active homogeneous fractions and determine their structures. XRD patterns lacked sharp Bragg reflections, indicating an amorphous (non-crystalline) structure consistent with many polysaccharides [23]. In the FT-IR spectra, the band at ~1598 cm^−1^ was assigned to the asymmetric stretching of carboxylate (COO^−^) groups from uronic acid residues (e.g., galacturonic and mannuronic acids) [24], suggesting that CDP is an acidic polysaccharide.

We systematically evaluated the anti-obesity effects of CDP in high-fat diet (HFD)-fed mice. Both the low-dose (SL) and high-dose (SH) CDP groups showed significantly lower body weight than the HFD group (*p* < 0.05), indicating that CDP attenuated HFD-induced weight gain. CDP also reduced relative adiposity, as reflected by decreases in inguinal (iWAT) and epididymal (eWAT) fat indices, supporting an anti-adiposity effect. These findings are consistent with prior reports that plant polysaccharides mitigate HFD-induced obesity in mice, including crude guava polysaccharides [25], and *Auricularia auricula* polysaccharides [26]. In the case of glucose tolerance, which reflects the capacity to maintain euglycemia after a glucose load [27], cocoa extract was able to improve glucose intolerance in mice on a high-fat diet [28]. Notably, the low dose (SL) produced a greater improvement than the high dose. Food and water intake were unchanged by CDP, excluding intake as a confounder. In serum, the high dose reduced total cholesterol (TC), triglycerides (TG), and low-density lipoprotein cholesterol (LDL-C), whereas the SL group showed no significant changes. In the liver, the SL group showed a significant reduction in TC. Taken together, these outcomes suggest that CDP may ameliorate lipid dysregulation—potentially through modulation of lipid absorption and metabolism, inhibition of fatty acid synthase activity, promotion of fatty acid oxidation, enhancement of antioxidant capacity, and regulation of genes such as PPAR-α [29]—although the precise mechanisms warrant further investigation.

To contextualize dose magnitude, we compared human-equivalent dose (HED) estimates with reported oral intakes of plant polysaccharides in humans (e.g., Lycium barbarum polysaccharides ≈ 150–300 mg·day^−1^; β-glucan ≈ 250–500 mg·day^−1^; certain Ganoderma extracts at gram-level intakes but with variable polysaccharide purity. Our HED totals—243–284 mg·day^−1^ (lower tier) and 486–568 mg·day^−1^ (higher tier)—situate the lower tier within the low-to-moderate intake range and the higher tier near or slightly above commonly reported upper bounds for β-glucan, while remaining well below gram-level whole-extract regimens. Given heterogeneity in source, polymer structure (molecular weight, branching, linkage configuration), and purity across preparations, these comparisons provide dose-magnitude calibration and feasibility context rather than claims of direct equivalence.

For intuition, oatmeal was used as a reference (1 serving = 40 g dry oats, providing ≈1.6 g soluble β-glucan). The human-equivalent, polysaccharide-normalized intakes correspond to 0.15–0.18 servings·day^−1^ (≈6–7 g dry oats) for the lower tier (0.243–0.284 g·day^−1^) and 0.30–0.36 servings·day^−1^ (≈12–14 g dry oats) for the higher tier (0.486–0.568 g·day^−1^). Assuming a polysaccharide content of 42.01 ± 0.45% (*w*/*w*, dry weight) in the finished CDP product, the corresponding finished product amounts are ≈0.58–0.68 g·day^−1^ (lower) and ≈1.16–1.35 g·day^−1^ (higher), with a relative uncertainty of ~±1.1% propagated from content variability. Practical unitization examples: 250 mg capsules (lower tier requires ~2–3/day [0.50–0.75 g]; higher tier ~5–6/day [1.25–1.50 g]) or 500 mg sachets (lower ~1–2/day [0.50–1.00 g]; higher ~2–3/day [1.00–1.50 g]); actual counts reflect rounding to whole units and thus modest deviation from targets. For consumer-facing formulations, once- or twice-daily administration with/after meals in ~150–200 mL warm water is reasonable; avoid prolonged heating and separate from oral medications by ≥2 h as a precaution. Real-world use should account for bioavailability and safety margins and does not constitute clinical dosing advice.

Histological assessment with hematoxylin and eosin (H&E) and Oil Red O staining revealed alterations characteristic of diet-induced obesity. H&E delineates tissue architecture and cellular pathology, whereas Oil Red O specifically labels neutral lipid deposits (e.g., lipid droplets). Diet-induced obesity is associated with adipocyte hypertrophy that facilitates excess energy storage and is linked to insulin resistance and local inflammation [30]. Consistent with prior evidence that plant polysaccharides can attenuate high-fat diet (HFD)-induced adipocyte expansion [31], we observed that HFD significantly increased the epididymal fat mass index and adipocyte size compared with the low-fat diet (LFD) group (*p* < 0.05). Notably, Coral dealbatus polysaccharide (CDP) reduced both parameters (*p* < 0.05). Chronic HFD feeding also led to hepatic steatosis, evidenced histologically by hepatocellular vacuolation/degeneration and abundant lipid droplets, whereas CDP administration markedly reduced hepatic lipid accumulation and ameliorated steatosis. Collectively, CDP mitigated epididymal adipocyte hypertrophy and reduced hepatocellular vacuolation and lipid deposition. These effects are consistent with mechanisms proposed in the literature—such as reduced hepatic fatty acid uptake and de novo lipogenesis—but were not directly assessed here.

Numerous studies have shown that a high-fat diet (HFD) induce obesity and perturbs the gut microbiota, and that host adiposity and the microbiota are bidirectionally linked. To probe potential mechanisms, we profiled the murine gut microbiota by 16S rRNA gene sequencing. CDP treatment significantly mitigated HFD-associated dysbiosis. Unexpectedly, Shannon diversity was significantly lower in the low-fat diet (LFD) group than in the HFD group, contrary to trends reported in many studies. A plausible explanation is that the relatively uniform carbon source in LFD simplified ecological niches and reduced diversity, whereas the higher diversity under HFD reflects stress-driven reorganization/expansion of pathobionts rather than a healthier state. At the genus level, the HFD group showed increased relative abundances of *Faecalibaculum* and *Odoribacter* compared with LFD; prior reports likewise describe elevated *Faecalibaculum* in HFD-induced obesity, with positive correlations to body-weight gain and hyperglycemia [32]. These expansions of putative pathobionts may partly account for the higher Shannon diversity observed in the HFD group. CDP significantly decreased the relative abundances of *Faecalibaculum* and *Odoribacter* and increased the abundance of beneficial taxa such as *Dubosiella*. *Dubosiella* has been reported to produce propionate and L-lysine, enhance mucosal barrier function, and modulate the Treg/Th17 balance to support intestinal homeostasis [33]; its abundance is negatively associated with body weight [34]. Thus, the CDP-induced rise in Dubosiella may contribute to the observed metabolic benefits under HFD.

LEfSe (linear discriminant analysis effect size) identifies taxa that differentially characterize groups [35]. In our dataset, LEfSe identified high-fat diet (HFD)-enriched discriminative taxa, including *Faecalibaculum*, *Odoribacter*, Marinifilaceae, Erysipelotrichaceae, Thomasclavelia, and Roseburia. Although *Roseburia* remained low in absolute abundance, it was relatively enriched in HFD; *Roseburia* is often regarded as a beneficial butyrate-producing genus implicated in metabolic and intestinal health, suggesting context-dependent shifts in our model. By contrast, Erysipelotrichaceae exhibited relatively high abundance, and elevated levels of this family have been associated with obesity, fatty liver, and atherosclerosis [36], consistent with reports linking it to metabolic and inflammatory dysregulation. CDP shifted these discriminant taxa toward low-fat diet (LFD)-like levels, supporting the interpretation that CDP mitigates HFD-induced obesity at least in part via modulation of the gut microbiota.

In summary, this study provides evidence that crude polysaccharides from *Coral dealbatus* (CDP) mitigate high-fat diet (HFD)–induced adiposity in mice and attenuate HFD-associated gut microbiota dysbiosis. Mechanistic elucidation was limited; nevertheless, to the best of our knowledge, this is the first evaluation of CDP for ameliorating HFD-induced obesity and dysbiosis, providing preliminary support for its relevance to functional-food development and metabolic-disorder research. Follow-up work will apply bioactivity-guided fractionation—graded precipitation, cellulose-based column chromatography, and gel filtration—to isolate highly active homogeneous fractions and elucidate their structures. Building on these fractions, we will interrogate mechanisms by profiling relevant signaling pathways and gene-expression programs to strengthen biological inference.

## 5. Conclusions

CDP mitigated high-fat diet (HFD)-induced excessive body weight gain and adiposity index in mice. It also improved glucose tolerance and reduced serum and hepatic levels of TC and TG. Furthermore, CDP attenuated lipid droplet accumulation in epididymal adipose and hepatic tissues and ameliorated gut microbiota dysbiosis in HFD-fed mice. Collectively, CDP reduced fat accumulation and improved gut dysbiosis induced by HFD.

## Figures and Tables

**Table 1:** Analysis of CDP monosaccharide composition.

Name	Peak Area	RT	Height	μg/mL
Mannose	193,400	13.077	12,486	6.128
Ribose	45,799	16.934	2283	1.166
Rhamnose	110,071	17.771	5267	3.977
Glucuronic acid	67,964	21.029	1650	2.534
Galacturonic acid	471,955	23.892	15,806	17.011
Glucose	1,368,637	27.746	41,659	45.197
Galactose	461,149	31.721	11,946	11.836
Xylose	38,190	33.411	1088	1.079
Arabinose	469,423	34.660	11,639	12.779
Fucose	16,604	39.841	310	0.537

**Table 2:** Results of KEGG difference analysis.

Pathway ID	Pathway Name			*p*-Value
HFD vs. LFD				
K03536	ribonuclease P protein component	0.078	0.095	<0.001
K07718	two-component system, sensor histidine kinase YesM	0.176	0.019	<0.001
K01934	5-formyltetrahydrofolate cyclo-ligase	0.078	0.096	<0.001
K02914	large subunit ribosomal protein L34	0.079	0.095	<0.001
K01783	ribulose-phosphate 3-epimerase	0.087	0.096	<0.001
K06178	23S rRNA pseudouridine2605 synthase	0.054	0.094	<0.001
K02078	acyl carrier protein	0.144	0.098	<0.001
K01653	acetolactate synthase I/III small subunit	0.102	0.085	<0.001
HFD vs. SL				
K10117	raffinose/stachyose/melibiose transport system substrate-binding protein	0.192	0.144	<0.001
K10119	raffinose/stachyose/melibiose transport system permease protein	0.163	0.114	<0.001
K00014	shikimate dehydrogenase	0.097	0.087	<0.001
HFD vs. SH				
K03522	electron transfer flavoprotein alpha subunit	0.086	0.013	<0.05
K00053	ketol-acid reductoisomerase	0.046	0.036	<0.05

## Data Availability

The original contributions presented in this study are included in the article. Further inquiries can be directed to the corresponding authors.
